# Editorial: Invertebrate neurophysiology—of currents, cells, and circuits

**DOI:** 10.3389/fnins.2023.1303574

**Published:** 2023-10-13

**Authors:** James M. Newcomb, Krista Todd, Edgar Buhl

**Affiliations:** ^1^Department of Biology and Health Science, New England College, Henniker, NH, United States; ^2^Neuroscience, Westminster University, Salt Lake City, UT, United States; ^3^School of Physiology, Pharmacology and Neuroscience, University of Bristol, Bristol, United Kingdom

**Keywords:** electrophysiological techniques, neuronal modeling, invertebrates, synapse, neural circuits

Exploring the intricacies of the brain and comprehending how it gives rise to behavior, still stands as one of the foremost challenges for the scientific community. To investigate the functioning of the nervous system directly, researchers have often employed electrophysiological techniques. Nonetheless, the complexity and sheer abundance of neurons, along with their myriad connections, present significant obstacles. Consequently, scientists have turned to the study of simpler organisms with intricate behavioral patterns but a substantially lower number of neurons. Thus, over the years, invertebrates have been extensively employed in neuroscience research owing to their relative simplicity, accessibility, and lower ethical concerns compared to vertebrate models. Their significant contributions and pioneering role in advancing our understanding of neuroscience are evident through the numerous discoveries made using these organisms. As a result, the central neural circuits of worms, molluscs, insects, and crustaceans were characterized well before similar findings emerged from vertebrate preparations, highlighting the extensive and valuable history of invertebrate research in neuroscience (Clarac and Pearlstein, 2007). These ground-breaking achievements have unraveled numerous fundamental mechanisms underlying neuronal function and this Research Topic comprising nine original research articles and four reviews, emphasizes the continuing breakthroughs made by researchers investigating invertebrates like molluscs, insects, crustaceans, and others.

Invertebrate nervous systems offer several advantages over their vertebrate counterparts for trying to investigate and understand a range of different neurobiological phenomena. These advantages include large, individually identifiable neurons in some species, such as molluscs (Croll, 1987; Bullock, 2000). These relatively large neuronal somata facilitate intracellular electrophysiology, dynamic clamp, cell isolation and culture, DNA/RNA sequencing of individual neurons, and much more (Katz and Quinlan, 2019). In this Research Topic, Zhuo et al. review many of these techniques used in the infamous *Aplysia californica* nervous system, including focused ultrasound, optical recording and stimulation, and especially the more recent infrared neural inhibition, spearheaded by these authors over the last decade. Also in this topic, Lee and Watson use a molluscan nervous system to better understand the neural mechanisms underlying modulation of feeding behavior in the nudibranch, *Melibe leonina*. This work on feeding complements prior work in this species, on identified neurons involved in locomotion (Thompson and Watson, 2005; Sakurai et al., 2014).

Having access to identifiable neurons has facilitated the study of central pattern generators (CPGs) in numerous invertebrates, including crayfish swimmerets, feeding and swimming in gastropods, the leech heartbeat and swimming, the stomatogastric ganglion of crustaceans, and walking in stick insects (reviewed in Marder et al., 2005; Selverston, 2010). In this topic, Pirtle reviews over four decades of research on the CPG controlling locomotion in the pteropod *Clione limacina*, particularly mechanisms that contribute to acceleration of swimming, which is achieved through neuromodulation of the swim CPG. Blitz reviews neuromodulation in a number of invertebrate CPGs, especially the circuits in the crustacean stomatogastric ganglion that are responsible for regulating gastric activity. Both of these reviews highlight many of the neuromodulatory principles that have been learned from these invertebrate preparations, including co-transmitters, the roles of neuromodulatory projection neurons, state dependence, the role of sensory feedback, communication between CPG circuits, and many others.

Another advantage to invertebrates for unraveling elements of the nervous system, is that invertebrate taxa cover a far wider phylogenetic spread than vertebrates. Therefore, comparing the nervous systems of phylogenetically disparate invertebrates can be very informative in learning about conserved neurobiological principles and evolution of nervous systems. In this topic, there are articles on a wide array of bilaterian invertebrates, including ecdysozoans (Arthropoda—Au et al.; Cillov and Stumpner; Au et al.; Blitz; Megwa et al.; Powell et al.) and lophotrochozoans (Mollusca—Lee and Watson; Pirtle; Zhuo et al.; Gribkova et al.). However, research on cellular signaling and neurons in metazoans that diverged from bilaterians can provide additional understanding about the evolution of nervous systems. Moroz et al. and Norekian and Moroz investigate the use of various peptidergic and nitrergic signaling compounds in ctenophores, which may have independently evolved a nervous “system” (Moroz et al., 2014; Moroz and Kohn, 2016; Burkhardt et al., 2023). Going even further afield in the metazoan clade, Nikitin et al. report on the use of amino acids in nerveless placozoans, to integrate various behaviors, such as feeding and locomotion. Ultimately, one of the goals of this type of research in these invertebrate systems is to determine if certain principles can be applied to other organisms, such as mammals. Here, Gribkova et al. attempt to do just that, pointing out similar parallels in modular arrangement of nervous systems between soft-bodied invertebrates and vertebrates. These similarities in arrangement suggest that continued work in these simpler circuits may shed interesting light on vertebrate nervous systems.

Insect preparations with their simple nervous systems with well-defined neural circuits have been instrumental to study the neural basis of complex behaviors observed in their natural environments, providing valuable insights into general principles of neural processing and decision-making (e.g., Menzel, 2012). For example, crickets, bush crickets, and grasshoppers exhibit highly specialized auditory sensory systems and central circuits, allowing them to engage in acoustic communication (Huber et al., 1989). Their relatively larger and fewer identifiable neurons facilitate the consistent identification of individual homologous neurons across different animals and species. Exploiting this unique advantage, Cillov and Stumpner review and describe some novel elements in local prothoracic auditory neurons, investigating the evolution of nervous systems and deepening our understanding of their development and function.

The circadian clock is another well-researched behavior in insects, and the fruit fly *Drosophila melanogaster* has played a crucial role in understanding the molecular clock over the past five decades since the discovery of the first clock mutant (Konopka and Benzer, 1971), as recognized by the Nobel Prize in 2017. In addition to its small size, short generation time, rapid life cycle, and easy breeding at a low cost, *Drosophila*'s simple and well-characterized genetics have made it an invaluable and highly tractable tool for studying neural function and behavior as well as human disease (Bier, 2005). Researchers use heterologous gene expression, utilizing the binary GAL4/UAS system from yeast to introduce genes from different species (Brand and Perrimon, 1993). By exploiting this genetic flexibility and the significant genetic similarities to other insects, such as mosquitoes, Au et al. revealed that the Cryptochrome 1 proteins from nocturnal and diurnal mosquitos can mediate distinct physiological and behavioral responses to blue light in flies, aligning with the specific behaviors of the different mosquito species. Consequently, the authors were able to manipulate the behavior of the fruit flies based on the mosquito version of the protein expressed. More recently, fruit flies have also become accessible for electrophysiological studies, particularly patch clamp recordings, enabling researchers to understand and define the clock circuit responsible for the fly's time perception using just 150 neurons. Notably, the light input and arousal LNv neurons are well-studied, and here Au et al., using a similar experimental approach to above, showed that different photoreceptor systems are integrated in the LNvs to provide functional redundancy for wavelength-dependent light perception that triggers behavioral arousal in the flies.

Lastly, since the seminal work of Hodgkin and Huxley in the 1950s (Hodgkin and Huxley, 1952), computational modeling in neuroscience has become increasingly important and impactful, especially with the ever-increasing computational power available to researchers. In this collection, utilizing computational modeling, Megwa et al. study the mechanism of the sodium potassium ATPase ion pump affecting neuronal plasticity in the well-established larval fly motoneuron system. Accomplishing this task experimentally would have been exceedingly challenging; moreover, its application to vertebrate systems is facilitated by the models' universality.

The objective of this Research Topic was to emphasize and advocate the valuable role of invertebrate preparations for neuroscience research. We received a diverse array of contributions studying a range of different species and techniques and encompassing both original research studies and comprehensive reviews on invertebrate electrophysiology. Our aim is to inspire and motivate more researchers to embrace and study a broad range of invertebrate species using a wide spectrum of electrophysiological techniques once again.

## Author contributions

JN: Conceptualization, Writing—original draft, review, and editing. KT: Writing—review and editing. EB: Conceptualization, Writing—original draft, review, and editing.
